# Immune Sera

**Published:** 1904-04

**Authors:** 


IMMUNE SERA. Hemolysius, Cytotoxus and Precipitus. By Prof. A. Wassermann, M.D., University of Berlin. Authorized translation by Chas. Bolduan, M.D., New York. John Wiley & Sons, 1904.

Serum diagnosis and therapy, which have engaged the attention of the profession to such a notable degree within the last few years, has not been systematically treated of in the English langauge hitherto. This little book endeavors to put briefly and succinctly the facts of the subject. The translation is faithful to the original, nothing but a table of contents having been added. The work is needed, and will fill a definite want.

